# Marketing of Foods to Children through Food Packaging Is Almost Exclusively Linked to Unhealthy Foods

**DOI:** 10.3390/nu11051128

**Published:** 2019-05-21

**Authors:** Živa Lavriša, Igor Pravst

**Affiliations:** Nutrition Institute, Tržaška cesta 40, SI-1000 Ljubljana, Slovenia; ziva.lavrisa@nutris.org

**Keywords:** food marketing, children, food packaging, nutritional composition, nutrient profiling

## Abstract

The nutritional composition of foods marketed to children is important, as it can significantly influence children’s preferences. The objective of this research was to evaluate the presence of child-oriented food products in the food supply and to investigate their nutritional composition. The sample included 8191 prepacked foods in the Slovenian food supply available in the market in 2015. The nutrient profile (World Health Organisation Regional Office for Europe nutrient profile model) of the products with child-targeted promotions was compared to the nutrient profile of those without child-targeted promotions. Food categories with the highest proportion of products with child-focused promotions were “Breakfast Cereals” (17%), “Chocolate and Sugar Confectionery” (15%) and “Edible Ices” (13%). Altogether, 93% of all products with child-focused promotions and 73% of products without such promotions were classified as “not permitted”. The proportion of “not permitted” foods was significantly higher in products with child-targeted promotions, compared with products without child-targeted promotions (*p* < 0.0001), and this trend was observed in a majority of food categories. To protect children from exposure to the marketing of foods with less favourable nutritional compositions, public health strategies should be focused also towards limiting promotions of unhealthy foods to children on product packaging, not only in media.

## 1. Introduction

Childhood obesity has become a significant public health concern and one of the major epidemiological challenges around the globe [1], including in Slovenia [2]. Food marketing is seen as an important factor influencing children’s food knowledge and preferences [3]. Frequent exposure to food promotions contributes to strong positive affect towards specific brands and products. This could be potentially harmful, with a negative impact on children’s diets and long-term health, especially if the promoted foods are energy dense and/or high in unfavourable constituents [4]. Targeted marketing, which is extensively used to advertise different food products to children, raises serious concerns, as children are an especially vulnerable population and are easily influenced and misled [5]. By promoting energy-dense foods, high in fat and sugar and low in micronutrients and fibre, the food industry is identified as an important contributor to the childhood obesity problem. Marketing of different unhealthy food products, to which children are exposed on daily basis, has a considerable impact in creating the so-called obesogenic environment [6,7]. 

It has been shown that foods advertised to children in different media are mostly products with less favourable nutritional compositions, which should be avoided in the context of a healthy diet [8,9,10,11,12]. Consequently, several countries have already adopted policies restricting children’s exposure to the marketing of less-healthy foods [13]. 

However, food marketing to children is not limited only to different media platforms but is also extensively present directly on the food packaging of products in the food supply. As many food choices are made directly at the point of sale, food packaging plays an important role in the consumer’s purchase decisions [14]. Food marketing expenditures in traditional media are declining [15], so the use of food packaging as a marketing tool has gained importance [16]. Package designs which target children usually use bright colours, childish lettering, cartoon characters, celebrity photos, sportspersons and references to fun and play [17,18,19,20], primarily to attract children’s attention. Such child-oriented products have a major impact on children’s brand associations and taste preferences and lead them to think such products are better, tastier and more fun. As a result, children are more likely to opt for such foods over foods in ordinary packaging [21,22,23]. It has long been known that children remember brands accompanied with cartoons or child-appealing elements better than those without the presence of such elements [24]. Theoretically, if a company wants to start building brand recognition and loyalty early, it could aim at children around three years old, as they are already able to recognise and remember brands. Unfortunately, products using such child-oriented marketing have been mostly recognised to have a poor nutritional composition and are usually high in undesirable nutrients [17,25,26]. Different nutrient profile models can be used to determine whether foods marketed to children have a susceptible nutritional composition or not. One of them is the nutrient profile, proposed by the World Health Organization, which was designed specifically for regulating marketing foods to children. Different countries are developing and adapting their own nutrient profiling models to best meet specific national issues and recommendations [27]. For example, Chile has been known for its extensive approach to child marketing regulation [28]; in a number of other countries, a variety of different approaches is also being investigated [29,30,31]. While details of the used nutrient profile models vary, it is very important that models used in regulations are adopted to the specific situation in each country [32]. 

While the extent of child-targeted advertising on food packaging has been investigated in some countries [17,25,26], data from European Union (EU) countries are very scarce [26]. Being a small Central-European country, bordering only other EU member states (Austria, Italy, Hungary and Croatia), with near-average gross domestic product (GDP) per capita and a high number of foods imported from other EU countries, Slovenia represents an excellent case country for such an evaluation. The food supply in Slovenia has been shown to be very comparable to other EU countries when considering the use of nutrition and health claims on prepacked foods [33,34]. Like in other countries in the region, there are currently no restrictions for child-targeted advertising on food packaging in Slovenia. In Slovenia, the marketing of foods to children is currently restricted only on television [35]. 

For the purpose of the present study, we evaluated the nutritional composition of foods included in this study by the WHO Regional Office for Europe nutrient profile model (WHO NP) [36]. This model was developed to support European countries when developing policies for restricting food marketing to children. In Slovenia, this model, with some country-specific modifications, has already been applied to regulate food marketing to children on television [37].

The objective of the present study is to evaluate the presence of child-oriented food products in the food supply and to investigate their nutritional composition using the WHO Regional Office for Europe nutrient profile model. 

## 2. Methods

### 2.1. Data Collection

A cross-sectional study was conducted in Slovenia between January and February 2015. Information on prepacked foods available in the Slovenian market has been collected since 2011 [38,39,40]. Data for 2015 was collected according to previously described methodology [39] and in line with the protocol developed within the Global Food Monitoring Initiative (GFMI) [41]. Sampling was performed by researchers of the Nutrition Institute in supermarkets of three major retailers (Spar, Hofer and Mercator), which cover the majority of the Slovenian market, and with the agreement of the retailers. Sampling was done in Ljubljana, Slovenia. Unique European/International Article Number (EAN) barcodes were used as product identifiers to accelerate the database’s formation and avoid duplicate entries. In agreement with retailers, all prepacked food products were systematically photographed in the store (they were not purchased) to record all data on the product, and all the data available on the packaging was recorded in an online Composition and Labelling Information System (CLAS) database [42], where data was extracted from the pictures. The database assembled in 2015 consisted of 10,674 food products. The information collected from food packaging included each product’s name, list of ingredients, nutritional values, packaging volume, price and EAN barcode. Details about this data collection were previously described in detail [40]. Additionally, information on child-oriented elements on food packaging was also extracted from photographs of all prepacked food products in the final sample. Food products from all available food categories were included for the study, except baby foods, food supplements, alcoholic beverages and flour and spices. The final sample contained 8191 unique prepacked food products with labelled nutritional composition. Foods without labelled nutritional composition were excluded from the study.

Products with children-targeted elements were identified according to criteria which have previously been described in other research [17,18,19,25]. These included use of promotional, licensed or cartoon characters; use of childish lettering; unusual shapes or bright colours referring to children, fun and/or play; cartoons; premium offers with toys, games and/or puzzles; and cross-promotions with children’s television programmes/films/cartoons/websites to attract children’s attention anywhere on the food packaging. Data was collected in a Microsoft^®^ Excel 2016 (16.0) (Redmond, WA, USA) spreadsheet for further analysis. 

### 2.2. Food Categorisation and Nutrient Profiling

Foods were categorised into food categories according to the WHO Regional Office for Europe nutrient profile model [36]. The WHO NP model was introduced as an answer to the adopted Vienna declaration on Nutrition and Noncommunicable Diseases in the Context of Health 2020 [43], which stated that “decisive action to reduce food marketing pressure to children with regard to foods high in energy, saturated fats, trans fatty acids, free sugars or salt” is needed. This model was developed as a common tool for use by governments in EU member states for the purpose of restricting food marketing to children and has been identified as a key activity of the European Food and Nutrition Action Plan 2015–2020 [44]. It was developed based on the Norwegian and Danish nutrient profile model as an answer to the emerging need to regulate food advertising to children. It is based on dividing foods into food categories rather than being a scoring system for specific nutrients. Such a model based on food categories is easier to adapt and modify for use in specific countries. The model divides foods into 17 different food categories, of which some categories are either “permitted” or “not permitted” for marketing to children by default, while others have a set of threshold limits for specific nutrients. Which foods belong to a specific food category is determined by using international customs tariff codes.

To complete nutrient profiling according to the WHO Regional Office for Europe nutrient profile model, the following data on nutritional composition was collected from food labels: fat, saturated fat, total sugars, added sugars, added sweeteners, salt and energy content. All food products in the sample were then subjected to nutrient profiling according to the WHO Regional Office for Europe nutrient profile model, accounting for the mentioned nutritional parameters. 

### 2.3. Data Analysis

Coding reliability was checked by a second coder to ensure intercoder reliability. Five percent of all food products with labelled nutritional compositions were checked for the presence of child-oriented elements to avoid discrepancies. Cohen’s Kappa was calculated (Kappa = 0.979), which showed very good agreement [45]. Discrepancies were discussed to ensure further coding consistency.

To illustrate significant differences between nutrient profiling outcomes between products without children promotions and products with children promotions (PCP), a chi-square test was utilised. *p* < 0.05 was considered statistically significant. For statistical analysis, XLSTAT 2017—Data Analysis and Statistical Solution for Microsoft Excel (Addinsoft, Paris, France) was used.

## 3. Results

Child-targeting elements on food packaging were found on 438 prepacked food products, which represented 5.3% of all food products in the dataset. The food category with the highest proportion of child-targeted promotions on the food packaging (Table 1) was “Breakfast Cereals” (17%), followed by “Chocolate and Sugar Confectionery” (15%) and “Edible Ices” (13%). 

**Figure 1:**
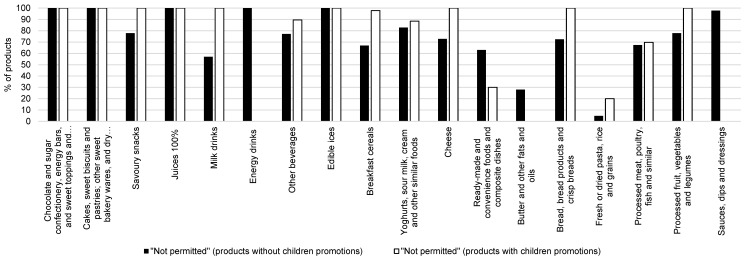
Proportion of products, scored as “not permitted” (by the World Health Organization Regional Office for Europe nutrient profile model) in specific food categories for products without children promotions (■black bars) and for products with children promotions (□white bars).

About 93% of products with child-targeted promotions and 73% of products without children promotions were identified as “not permitted” according to the WHO NP model. However, most products with children promotions identified as “permitted” were from the categories of “Processed Meat, Poultry, Fish and Similar”, “Ready-Made and Convenience Foods”, “Other Beverages” and “Fresh or Dried Pasta, Rice and Grains”. Figure 1 shows that, compared with products without children promotions, products with children promotions were more likely to have a higher proportion of not permitted foods within the categories (*p* < 0.0001). In most food categories, foods with children promotions more often scored as “not permitted”; the only exception with an opposing ratio was the category of “Ready-Made and Convenience Foods”. This phenomenon was particularly notable in “Milk Drinks”, “Breakfast Cereals”, “Cheese” and “Bread, Bread Products and Crisp Breads”. Differences between nutrient profiling outcomes between products with and without child-targeted promotions were statistically significant in “Savoury Snacks”, “Breakfast Cereals”, “Ready-Made and Convenience Foods and Composite Dishes” and “Processed Meat, Poultry, Fish and Similar”.

## 4. Discussion

The results of the present study showed that most prepacked foods in Slovenia have a poor nutritional composition. The percentage of “not permitted” products, according to the WHO nutritional profile, is higher for foods with children promotions. These results are comparable with other studies, however, these were conducted outside of Europe in countries with considerable differences in food regulation and traditions, which might affect the food supply [17,18,25,46,47]. In Europe, Lythgoe et al. [26] investigated the nutritional composition of foods marketed to children and reported that a significant number of foods with such children-targeted elements on the packaging were higher in fat, sugar and salt in comparison with foods targeted to the general population. However, they did not conduct nutrient profiling; therefore, the results of both studies cannot be directly compared.

The reported results are of great concern because it is well established that different child-oriented marketing techniques, such as the use of cartoon characters and similar elements on food packaging, significantly influence children’s food choices and preferences [21,23,48]. By using such child-oriented marketing techniques, children are encouraged to like and want products which they see as “fun” and “good tasting” [49]. When foods are accompanied with children-familiar characters, children are convinced it tastes and looks better and would more likely ask their parents to buy it for them [50]. If these products have poor nutritional compositions, this can significantly affect their future dietary habits and health. It has been also shown that preschool children are especially highly influenced by different social factors and prefer not only foods which their friends like but also foods which are presented as “for children” and are accompanied by positive and child-targeted elements [51]. In general, we noticed that products with children promotions had a higher proportion of products which were identified as “not permitted” than products without child-targeted promotions and, therefore, had less favourable nutritional compositions than those without children promotions. Such a phenomenon has also been noticed in some other studies [18,26,52]. Child-targeted products also tend to use characters familiar to children and, consequently, children prefer such products, despite their usually poor nutritional composition [53]. The category of “Breakfast Cereals”, for example, which was the category with the highest number of products with children promotions, had significantly (*p* < 0.0001) more products which scored as “not permitted” than products from the same category with no children promotions. Among products with child-targeted promotions in the “Breakfast Cereals” category, only 2% scored as “permitted”. As other studies have suggested, this food category is one of the most problematic—breakfast cereals with children promotions had higher than average levels of sugar and refined grains [54], contained less fruits and nuts and were overall less nutritious than adult-targeted cereals [52]. 

Although we found some products with children promotions that were identified as “permitted” according to the WHO nutrient profile model, these were mostly products which should be, if following dietary guidelines for children [55,56], consumed in moderation, despite passing the criteria for permitted advertising. These were products such as salamis, hot-dogs, fruit yoghurts, meat pates and instant soups. However, it is encouraging that we also found children promotions on some healthy products, for example, on natural mineral water, instant wheat grits without added sugar and plain pasta with natural vegetable extracts. The outcomes of nutrient profiling can vary depending on which profiling model is used [57]. It is important for regulating purposes that the model is carefully chosen, as it can have significant policy as well as public health consequences [32]. In our study, we chose the WHO NP model, as this model was already used in Slovenia, as a basis for regulation of marketing foods to children on television. Also, this model is currently the one which was proposed to harmonise regulation of food marketing to children in EU countries. A comparison of different profile models showed that the WHO NP model is not among the strictest models to regulate food marketing to children [32]. As this model permits or excludes certain food categories from marketing to children by default, it is a question if such evaluation can be a reasonable system for regulation. It is also more difficult for consumers to choose healthier options in permitted and not permitted categories. However, some modifications of the WHO NP model were already applied in Slovenia when setting guidelines for regulation of food marketing to children on television [37]. 

A major strength of the present study is that we operated with an extensive data collection, which covered the majority of the Slovenian market. The database compiled is also useful for other purposes involving studying the nutritional composition of foods, as well as for monitoring changes in the food supply over the years. While a large database allowed a reliable assessment of marketing on food products, it also meant an extensive number of products and data needed to be collected. Data analysis was conducted in one replication, but the validity of the results was confirmed on a subsample of products. A limitation of the study was also that some food categories were excluded from analysis, but it should be noted that we only excluded those categories where we did not expect any child-focused marketing. An exception was food supplements, which were excluded because the sample was collected in food stores, which in Slovenia have a very limited selection of food supplements (which are mostly sold in pharmacies and specialised stores). Therefore, the results for the category of food supplements would not be representative of the whole market. The results apply for the year 2015 and conditions on the market could have changed since then. As currently we do not have any evidence to support these changes, further studies of these will be needed in the future to track changes on the market.

**Figure 2:**
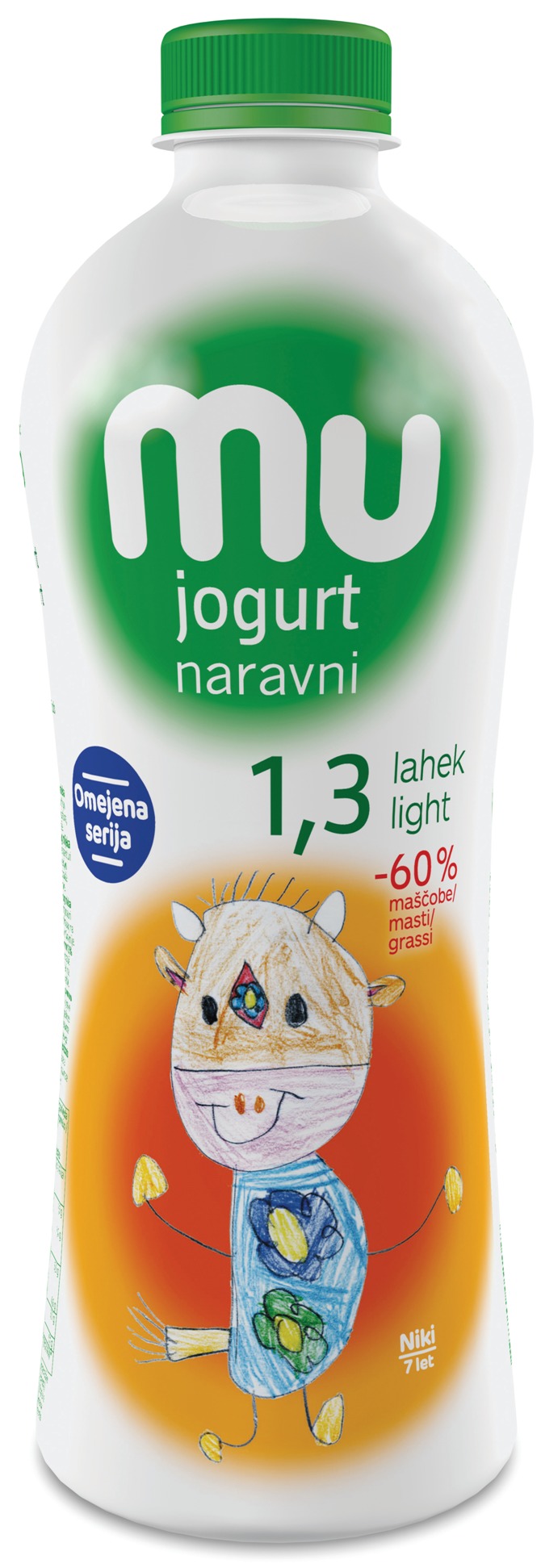
Example showing that child-targeted marketing can be also used for promotion of minimally processed foods: packaging of plain yoghurt with reduced fat.

Current evidence shows a worldwide trend of food products with children promotions being predominantly those with less-favourable nutritional compositions. As children can be easily influenced by marketing, the industry should be encouraged to use such child-oriented promotions on foods with more favourable nutritional compositions, rather than on those which are considered less healthy. A number of studies showed that child-oriented promotions on food packaging are effective on healthy foods [23,50,58,59]. For example, children were more likely to choose a healthy food product when it was accompanied by a child-appealing character than a healthy product without it. Adding such a character resulted in more willingness to choose and eat such food and also in higher wished-for frequency of consumption [59]. Encouraging children to choose healthier dietary options is necessary for promoting a healthy lifestyle and dietary patterns. Using child-oriented marketing on healthy foods could make an important contribution to children’s diets overall. There are examples showing that the food industry is already using child-oriented marketing for the promotion of minimally processed foods (for example, natural yoghurt with reduced fat; see Figure 2). However, it remains to be elucidated why such marketing techniques are mostly used on foods with less-favourable nutritional compositions. 

Currently, advertising foods to children is poorly regulated in many countries. In Slovenia, regulation of television marketing was implemented in 2017, but our recent research shows that there is a need for such regulation on other media platforms [60]. Results from this study suggest that public health interventions should not be focused only on the marketing of less-healthy foods in media but should also target other factors, such as child-oriented marketing on food products, which is one of many factors that contribute to an unhealthy obesogenic environment for children. Given the concern about childhood obesity and the vast burden of noncommunicable chronic diseases [61], more focused and effective public health strategies are needed.

## 5. Conclusions

The results of our study show that foods with child-targeted promotions were more likely to score as “not permitted” for children marketing (based on nutrient profiling by the WHO NP), compared to products without child-targeted promotions (*p* < 0.0001). This trend was observed in most food categories. To protect children from exposure to the marketing of foods with less favourable nutritional compositions, public health strategies should be focused also towards limiting promotions of unhealthy foods to children on product packaging, not only in media.

## Figures and Tables

**Table 1:** Number of products with labelled nutritional compositions and proportion of products with child-targeted promotions.

WHO ^1^ Category		*N* of All Products with Labelled NC ^2^	PCP ^3^ (%)
1	Chocolate and sugar confectionery	1228	15%
2	Cakes, sweet biscuits and pastries	697	5%
3	Savoury snacks	339	6%
4a	Juices 100%	149	5%
4b	Milk drinks	178	3%
4c	Energy drinks	25	0%
4d	Other beverages	748	5%
5	Edible ices	190	13%
6	Breakfast cereals	272	17%
7	Yoghurt and fermented milk	566	5%
8	Cheese	358	1%
9	Ready-made and convenience foods	678	1%
10	Butter and other fats and oils	239	0%
11	Bread, bread products and crisp breads	241	0%
12	Fresh or dried pasta, rice and grains	506	1%
13	Fresh and frozen meat, poultry, fish and similar	106	0%
14	Processed meat, poultry, fish and similar	655	5%
15	Fresh and frozen fruit, vegetables and legumes	101	0%
16	Processed fruit, vegetables and legumes	648	0%
17	Sauces, dips and dressings	267	0%

^1^ WHO—World Health Organization, ^2^ NC—nutritional composition, ^3^ PCP—products with children promotions.
